# Supplementary material for: The Construction and Exploration of a Comprehensive MicroRNA Centered Regulatory Network in Foxtail Millet (Setaria italica L.)
Source: Front Plant Sci. 2022 May 6;13:848474. doi: 10.3389/fpls.2022.848474 (PMC9121102; doi:10.3389/fpls.2022.848474)
Supplement: Supplementary file 7 [file Data_Sheet_7.zip › Sit-miRN3002_Seita.3G151000.1_1947_TPlot.pdf]

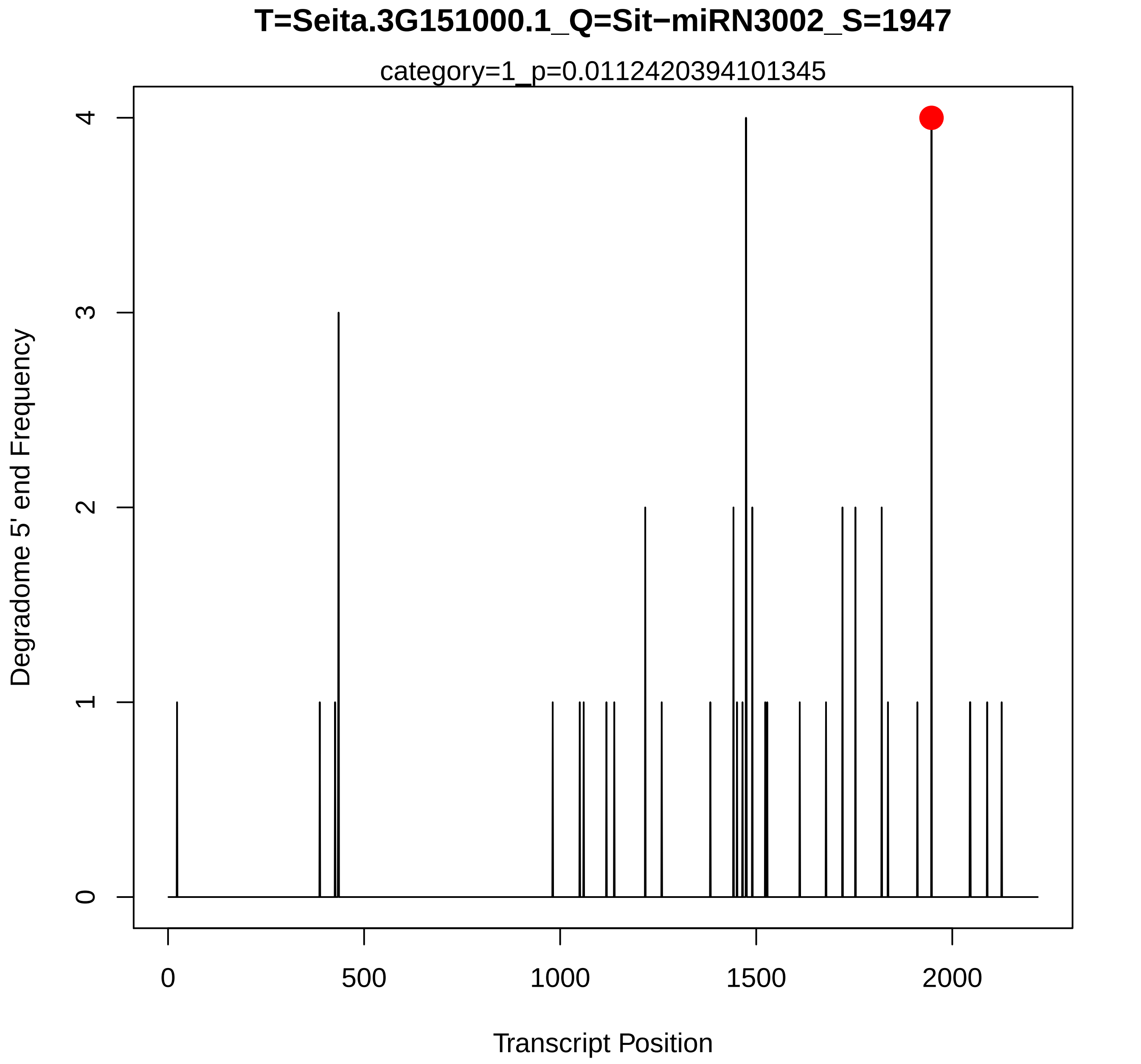
T=Seita.3G151000.1_Q=Sit−miRN3002_S=1947
category=1_p=0.0112420394101345
Degradome 5' end Frequency
0
1
2
3
4
0
500
1000
1500
2000
Transcript Position
